# Orosomucoid-1 Expression in Ameloblastoma Variants

**Published:** 2016

**Authors:** Alejandro García-Muñoz, Ronell Bologna-Molina, Mario A. Rodríguez, Rodrigo Liceága-Reyes, Jose Eduardo Farfán-Morales, Saray Aranda-Romo, Nelly Molina-Frechero, Rogelio González-González

**Affiliations:** 1 *Laboratorio de Investigación en Odontología, ALMARAZ/UBIMED, FES Iztacala, UNAM. State of Mexico, Mexico.*; 2 *Molecular Pathology, Universidad de la República (UDELAR), Montevideo, Uruguay.*; 3 *Department of Infectomics and Molecular Pathogenesis, CINVESTAV-IPN, México, D.F, México.*; 4 *Maxillofacial Surgery Department. Hospital Juárez de México, México D.F, México.*; 5 *Instituto Nacional de Pediatría, México D.F, México.*; 6 *Biochemistry, Microbiology and Pathology Laboratory, Faculty of Stomatology, Universidad Autónoma de San Luis Potosí, San Luis Potosí, Mexico.*; 7 *Health Care Department, Universidad Autónoma Metropolitana, Xochimilco, UAM, México City, Mexico.*; 8 *Department Research, School of Dentistry, Universidad Juárez del Estado de Durango, México.*

**Keywords:** Orosomucoid-1, ameloblastoma, ameloblastic carcinoma, odontogenic tumors

## Abstract

Odontogenic tumors constitute a group of heterogeneous lesions of benign and malignant neoplasms with variable aggressiveness. Ameloblastomas are a group of benign but locally invasive neoplasms that occur in the jaws and are derived from epithelial elements of the tooth-forming apparatus. We previously described orosomucoid-1 protein expression in odontogenic myxomas. However, whether orosomucoid-1 is expressed in other odontogenic tumors remains unknown. Since orosomucoid-1 belongs to a group of acute-phase proteins and has many functions in health and disease, we identified and analyzed orosomucoid-1 expression in ameloblastoma variants and ameloblastic carcinoma using western blot and immunohistochemical techniques. Thirty cases of ameloblastoma were analyzed for orsomucoid-1; five specimens were fresh for western blot study (four benign ameloblastomas and one ameloblastic carcinoma), and 25 cases of benign ameloblastoma for immunohistochemical assays. Orosomucoid-1 was widely expressed in each tumor variant analyzed in this study, and differential orosomucoid-1 expression was observed between benign and malignant tumor. Orosomucoid-1 may play an important role in the behavior of ameloblastomas and influence the biology and development of the variants of this tumor.

Odontogenic tumors (OTs) constitute a heterogeneous group of relatively rare benign and malignant neoplasms that display variable aggressiveness (1,2). Ameloblastoma is a benign but locally aggressive OT of the mandible and maxilla that could cause severe facial disfigurement and functional impairment if neglected (3). Ameloblastoma is thought to arise from epithelial cells in developing teeth, including cells of the dental lamina and enamel organ (4). However, the molecular mechanisms that regulate ameloblastoma cell growth and invasion are unknown. In Mexico, the prevalence of ameloblastoma has been estimated to be 23.7% of all OTs (5). According to the World Health Organization (WHO) histological classification of tumors of 2005, ameloblastomas are classified into four variants: solid multicystic (SMA), extraosseous peripheral (PA), desmoplastic (DA) and unicystic (UA) (1). SMA and UA are the most common subtypes. UAs present some characteristics of odontogenic cysts; they are less aggressive than SMAs and tend to occur at an earlier age (6-8). Ameloblastic carcinoma (AC) is a very rare epithelial odontogenic malignancy characterized by cytological atypia and malignant behavior.

Orosomucoid-1 (ORM1) is a 41- 43- kDa glycoprotein with a pI of 2.8 to 3.4 that is produced in the liver and secreted into the serum during acute inflammation. ORM1 was described in 1956 by Schmid as a member of a group of acute-phase proteins that might play a role in modulating immune responses to stress, among other functions (9).

Several studies in the literature have described the use of ORM1 in the diagnosis of different cancer types such as bladder, colorectal, or ovarian cancer (10, 11).

ORM1 might play a role in defense or resistance mechanisms against tumor cells specifically by reducing the proliferation, invasion, and metastasis of cancer cells, thereby influencing tumor invasion and growth (11). ORM1 inhibits polymorphonuclear neutrophil activation and is therefore considered as a natural immunom-odulatory, anti-inflammatory, anti-neutrophil, and anti-complement agent (12).

ORM1 may also suppress lymphocyte and platelet responsiveness by interfering in a common activation pathway. ORM1 may perturb the lymphoid cell surface and interfere with events required for lymphocyte proliferation by altering membrane fluidity and inhibiting concanavalin A receptor and surface immunoglobulin capping (13). Additionally, in a study on human myocardial infarction, human polymorphonuclear cells were shown to synthesize and release ORM1, suggesting that ORM1 provides endogenous inhibitory feedback in response to excessive inflammation (14).

We previously reported that ORM1 is overexpressed in odontogenic myxomas (15). However, whether ameloblastic tumors express ORM1 remains unknown. We now consider this possibility because the particular characteristics of this tumor type such as growth and vascularization are consistent with those seen in other tumors in which ORM1 has been found. The aim of this study was to identify and to characterize the immunohistochemical expression pattern of ORM-1 protein in UA, SMA and malignant counterpart AC; to better understand the differences in the biological behavior of these tumors.

## Materials and methods

**Tissue samples and** **tissue preparation**

The Department of Maxillofacial Surgery of Juárez de México Hospital provided the tissue samples and the Research and Ethics Committee of this institution under the registration number HJM 1996/11.03.08 approved the research protocol.

A total of 30 OT cases were analyzed; five were fresh OT specimens (two UAs, two SMAs and one AC) that were removed during surgery and promptly frozen in liquid nitrogen before being stored at -70°C prior to western blot analysis. The other 25 ameloblastoma samples (13 UAs and 12 SMAs) were obtained from previously paraffin-embedded tissue blocks and were used for immunohistochemical assays. 

**Protein extraction**

The protein extraction protocol was adapted from the selective extraction method, with minor modifications (16). Briefly, samples were rinsed in commercial physiological solution, frozen in liquid nitrogen, mechanically pulverized and resuspended (400 mg tissue/ml) in sample buffer (urea 7 M, thiourea 2 M, CHAPS 4%, IPG buffer 2%, DTT 40 mM and milliq water) and complete™ protease inhibitor cocktail (Roche, Germany). Resuspended samples were sonicated on ice, insoluble material was removed by centrifugation (20,000 ×g, 5 min at 4 °C), and the supernatant was preserved. Additionally, we used a 2D Clean-Up Kit (Amersham Biosciences, USA) to clean the proteins. The precipitate was diluted in rehydration stock solution (7 M urea, 2 M thiourea, 2% (w/v) CHAPS, 0.5% (w/v)). 

**Western blot**

Western blot analysis was performed as previously described (17). Briefly, the proteins in rehydration solution were separated by 10% SDS-PAGE and transferred to nitrocellulose membranes. After blocking in 5% nonfat milk in Tris-buffered saline containing 0.05% Tween-20 (TBS-T) for 2 h, membranes were incubated with a primary antibody against ORM1 (1:5000) (Abcam) and then with an anti-mouse secondary antibody conjugated to horseradish peroxidase (Zymed) (1:10,000). An antibody against α-actin was also used, as an internal control. Finally, antibody detection was performed by chemiluminescence (ECL, GE Healthcare).

**Histochemical and immunohistochemical staining**

Ameloblastoma specimens were fixed in 10% buffered formalin and embedded in paraffin. To analyze the morphological characteristics of each neoplasm, samples were sectioned, mounted on microscope slides, stained with hematoxylin and eosin and examined by optical microscopy (Nikon H550 L, Yokohama, Japan). All slides were reviewed by an experienced pathologist for the histopathological classification of OTs according to the recent WHO classification of head and neck tumors (1).

Immunohistochemical studies were performed as previously described (18): 2-µm-thick sections were treated with 0.1 M sodium citrate (pH 6.2) and Tween-20 for epitope separation. Endogenous peroxidases were blocked with 0.9% hydrogen peroxide, followed by incubation with 1% bovine serum albumin (BSA) in phosphate-buffered saline (PBS) for 5 min to eliminate non-specific binding. Sections were then incubated with a primary monoclonal antibody against ORM1 (Abcam, dilution 1:70) for 45 min, followed by incubation with a biotinylated antimouse/antirabbit antibody and a streptavidin/peroxidase complex (LSAB+ Labeled Streptavidin-Biotin, Dako Corporation, Carpintería, CA, USA) for 30 min each. Reaction products were visualized with 3,3´-deaminob-enzidine -H_2_O_2_ substrate (DAB; Dako Corporation, Carpintería, CA, USA). Sections were countersta-ined with Mayer’s hematoxylin solution. PBS was applied as a substitute for the primary antibodies for the negative control, and an internal control was used for the positive control.

All cases with immunostaining between 10 to 50% were regarded as positive, and all cases with staining>50% were considered highly positive (19).

## Results

**ORM1 expression in ameloblastoma variants and ameloblastic carcinoma**

**Fig. 1:**
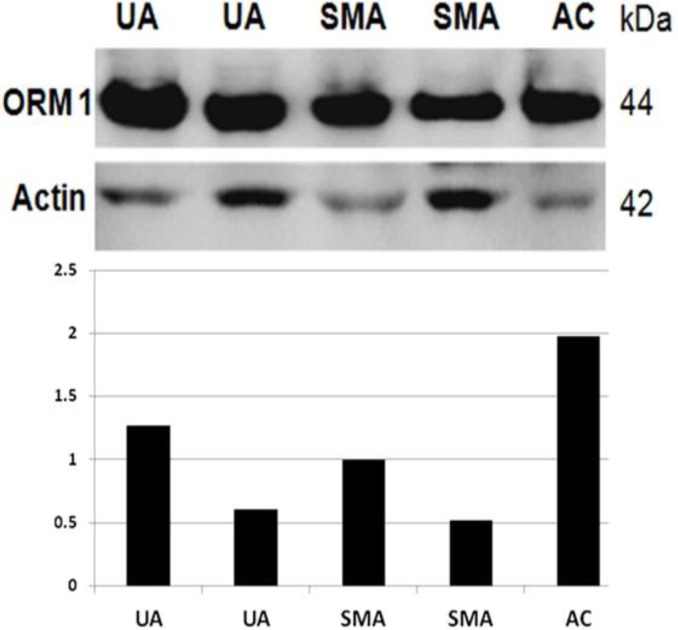
**ORM1 expression in ameloblastoma variants and ameloblastic carcinoma. **Representative Western blot assays are shown. Bands recognized by the ORM1 antibody were analyzed by densitometry, and the values were normalized with those obtained from the bands recognized by the actin antibody. The relative ORM1 expression in SMA samples was arbitrarily assigned a value of 1. UA: unicystic ameloblasoma; SMA: solid multicystic ameloblasoma; AC: ameloblastic carcinoma

To identify ORM1 protein in tumor samples, we performed Western blot assays using a commercial monoclonal antibody. The antibody strongly recognized a single protein band of approximately 44 kDa, which was the expected molecular weight of ORM1 in all the analyzed samples (Fig. 1). An intense 42-kDa band was also detected in all samples by an anti-actin antibody, which was used as an internal loading control (Fig. 1). We then performed densitometry of the bands detected by the antibodies to semi-quantitatively analyze the relative ORM1 expression in the samples. Interestingly, ORM1 levels were up to four times higher in AC than in UA or SMA (Fig. 1). Furthermore, ORM1 was expressed in all of the analyzed samples, but at varying levels.

**Confirmation and in situ determination of ORM1 expression in ameloblastoma variants**

**Fig. 2:**
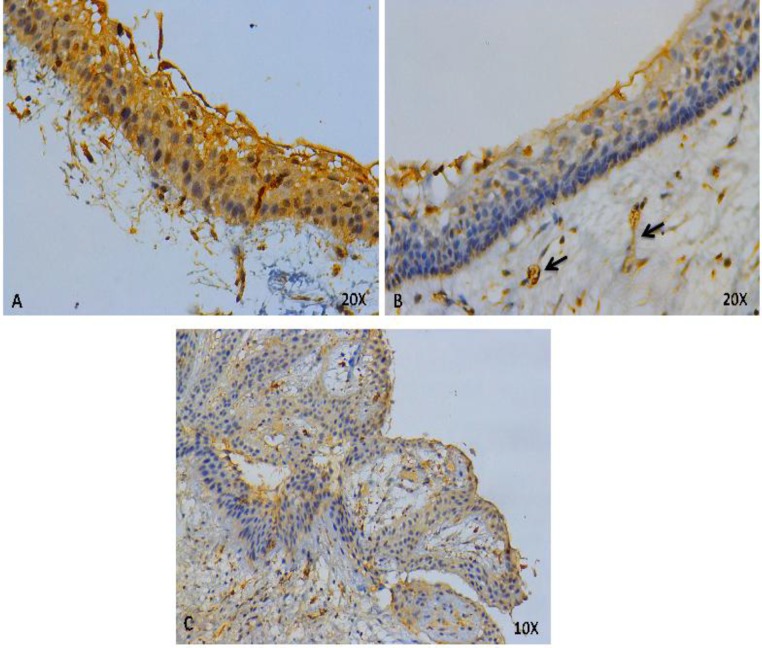
**ORM1 detection by immunohistochemistry in unicystic ameloblastomas**

**Fig. 3:**
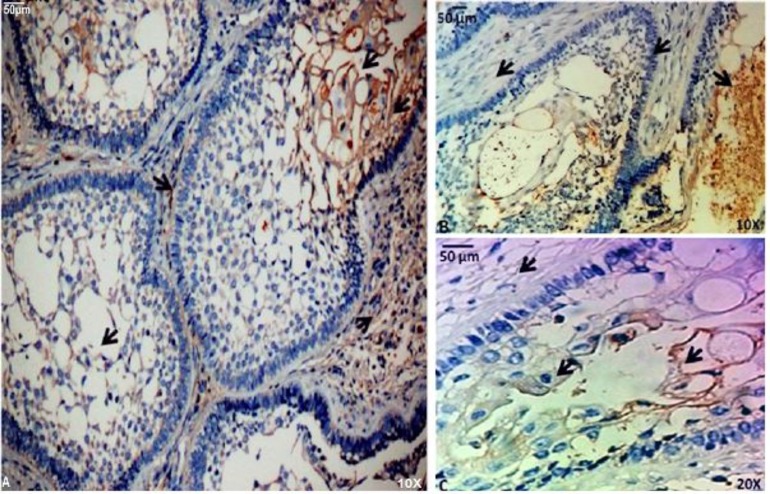
ORM1 detection by immunohistochemistry in solid multicystic ameloblastoma. (A) ORM1 was expressed in cells around and within microcysts, but little expression was observed in the cytoplasm of palisade cells (magnification x100). (B-C) ORM1 expression was strongly positive in microcysts, indicating that the benign cells secreted ORM1. A positive reaction was also found in stromal cells (magnifications x100 and x200). The arrows show expressions of ORM-1 in stroma and microcysts (A, B, C) and weak expression is observed in palisade cells (B

**Fig. 4:**
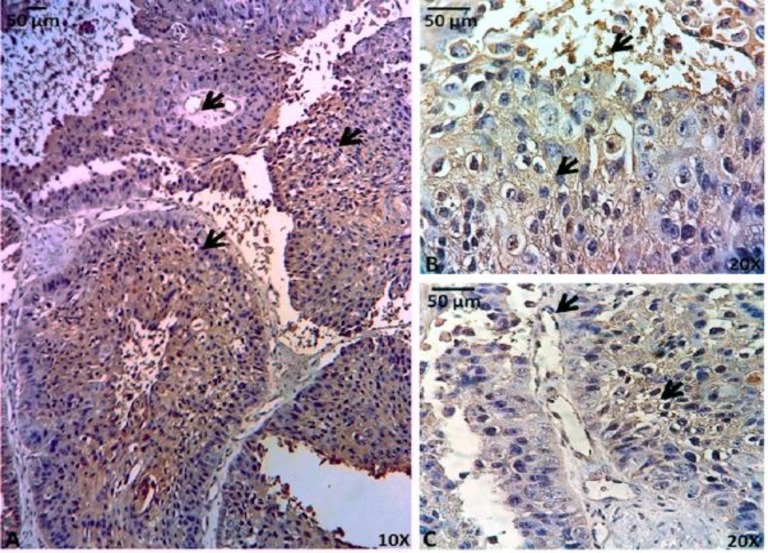
ORM1 detection by immunohistochemistry in ameloblastic carcinoma. *In situ* ORM1expression was observed in AC, indicated by the widely distributed and strongly positive reaction to the antibody against ORM1. Diverse ORM1 distribution was observed between pleomorphic cells, mitotic cells, cells with hyperchromatic nuclei, cells with inverted nuclear polarity and cells within and outside of microcysts. **(A)** ORM1 staining was positive in all cells (magnification x100) and microcysts (arrow). **(B)** Pleomorphic cells and cells in microcysts were also positive for ORM1 (arrow) (magnification x200). **(C)** The blood vessels and cells around microcysts were also positive for ORM1 (arrow) (magnification x200

To determine the *in situ* ORM1 expression pattern in clinically obtained tumor samples, we performed immunohistochemical assays using a monoclonal ORM1 antibody. ORM1 was expressed in all analyzed samples, particularly in the cytoplasm of epithelial tumor cells, in microcysts from some variants, within the endothelial cells of large and small blood vessels of all samples and within connective tissue stroma of AC and SMA only (Fig. 2, 3, 4).

**Table. 1:** Immunohistochemical quantification

**Tumor (n)**	**<50** **n(%)**	**>50** **n(%)**
SMA (14) UA (15) AC (1)	5(35.7) 8(53.3) ---------	9(64.2) 7(46.6) 1(100)

This table shows the quantification of the positivity for ORM1 by immunohistochemistry. All cases with immunostaining between 10 to 50% were regarded as positive, and all cases with staining > 50% were considered highly positive. More SMA cases had high positivity (> 50%) than UA case, whereas the only AC case was highly positive. UA: unicystic ameloblasoma; SMA: solid multicystic ameloblasoma; AC: ameloblastic carcinoma

Differences in ORM1 expression revealed that AC expressed ORM1 in more sites within neoplasms and that ORM1 is more abundant in AC than in SMAs and UAs (Table 1).

Interestingly, ORM1 was expressed in pleomorphic cells in some AC microcysts (Fig. 4A, B). Additionally, UAs expressed ORM1 in epithelial cells and blood vessels but not in stromal.

cells; in contrast, the stroma of ACs and SMAs expressed ORM1. Among these cases, SMAs contained more ORM1-expressing cells (Fig. 3A, 4A). Finally, the accumulation of ORM1 in some spaces within SMA microcysts suggests that ORM1 secretion might occur from these structures (Fig. 3A, B). Notably, due to the small number of cases (justified by the rarity of the tumors), only the percentages were described and considered as a trend (Table 1).

## Discussion

The acute-phase response is the reaction of an organism to a disturbance in homeostasis and is characterized by dramatic changes in the concentrations of certain plasma proteins, defined as acute-phase proteins (20). ORM1 is an acute-phase protein, and increased ORM1 levels have been reported in the serum of patients with various malignant diseases, including hepatocellular carcinoma, gynecological carcinomas, esophageal cancer and head and neck cancers (21-24). Human hepatocytes, endothelial cells and other cells normally produce ORM1 (21, 25, 26).

In the present study, ORM1 was expressed in all samples analyzed, suggesting an important role for ORM1 in the development and biological behavior of these tumors. In general, the distribution of ORM1 was the same in all analyzed samples (Fig. 2, 3, 4). However, in UAs, ORM1 was not as clearly expressed in the mesenchymal tissue (Fig. 2A, B). Moreover, all blood vessels were positive for ORM1.

Suggesting the specific function of ORM1 in ameloblastoma is difficult due to the multiple roles for ORM1 that have been described (9). The high expression of ORM1 can act as a defense mechanism against tumor cell proliferation and invasion. This mechanism has been suggested in colon cancer cells, in which ORM1 overexpression decreased the colony-forming capacity, invasion and adhesion, whereas ORM1 inhibition using antisense oligodeoxynucleotides increased these events (11)**.** Alternatively, given its anti-inflammatory activity, ORM1 overexpression could inhibit the immune response, resulting in increased tumor cell proliferation (9). ORM1 may also play an important role in angiogenesis (25), proliferation, invasion and adhesion (11), in the regulation and activation of immune cells, and finally, as a carrier of defense substances (9).

Thus far, background ORM1 expression has not been found in any variant of ameloblastomas and has only been found in odontogenic myxomas (15), but the nature of these tumors is different; ameloblastoma is an epithelial tumor, whereas odontogenic myxoma is a mesenchymal tumor.

It is generally considered that ORM1 is able to inhibit polymorphonuclear neutrophil activation, since it acts as a natural anti-inflammatory, anti-neutrophil, anti-complement and immunomod-ulatory agent (12). This leads us to consider a possible immunomodulatory function of ORM1 in the biological behavior of ameloblastomas.

On the other hand, there have been reports stating that ORM-1 by itself increases migration, but not proliferation of human dermal microvascular endothelial cells. Nevertheless, endothelial cells, in the presence of ORM1, are capable of stimulating the development of endothelial tubes (27). All this suggests that ORM1 is involved in the regulation of the angiogenic process (26, 27). Irmak et al. (10) explained that the highest increase of ORM-1 levels in the advanced phases of urinary bladder cancer, (belonging within the group of vascularized tumors), could partly be assigned to the production of ORM by the high number of endothelial cells of angiogenically active blood vessels. Ligresti et al. (28), supporting the concepts mentioned above, found that ORM1 is capable of potentiating the angiogenic effect of VEGF in endothelial cells in culture. Cultures treated with both these proteins showed an even greater number of vessels than cultures treated solely with VEGF (28).

In a recent work, our group has determined by immunohistochemical technique the presence of VEGF and ORM1 in odontogenic myxomas (29). All the previously gathered data suggest a possible collaborative pro-angiogenic role of ORM1, but to corroborate this possible function of ORM1 in ameloblastomas, it is necessary to perform other experimental approaches and functional assays, focused on the elucidation of how these proteins may cooperate directly in the growth of this tumor. In the present study, we report for the first time that ORM1 is expressed in the neoplastic tissue of benign and malignant ameloblastic tumors. Although we now have some information on the role of ORM1 in ameloblastoma and AC, many questions remain to be resolved. For example, although ORM1 expression was found in the analyzed samples, whether ORM1 expression was induced or merely increased in response to tumor growth or the onset of neoplastic cell production remains unknown. Additionally, the acidity and other properties of ORM1 may contribute to tissue degradation, thereby easing tumor growth.

Finally, although ORM1 is known to have many functions, immunological and functional experiments such as proliferation, migration and invasion assays or gene silencing and over-expression assays will be necessary to better elucidate the role of ORM1 in ameloblastomas.

## References

[1] Kleihues P, Sobin LH, Barnes L, Eveson JW, Reichart P (2005). Pathology and genetics head and neck tumors. World health organization classification of tumors.

[2] Bologna-Molina R, Mosqueda-Taylor A, Lopez-Corella E (2008). Syndecan-1 (CD138) and Ki-67 expression in different subtypes of ameloblastomas. Oral oncology.

[3] Sham E, Leong J, Maher R (2009). Mandibular ameloblastoma: clinical experience and literature review. ANZ journal of surgery.

[4] Heikinheimo K, Jee KJ, Niini T (2002). Gene expression profiling of ameloblastoma and human tooth germ by means of a cDNA microarray. Journal of dental research.

[5] Mosqueda-Taylor A, Ledesma-Montes C, Caballero-Sandoval S (1997). Odontogenic tumors in Mexico: a collaborative retrospective study of 349 cases. Oral surgery, oral medicine, oral pathology, oral radiology, and endodontics.

[6] Ueno S, Nakamura S, Mushimoto K (1986). A clinicopathologic study of ameloblastoma. Journal of oral and maxillofacial surgery : official journal of the American Association of Oral and Maxillofacial Surgeons.

[7] Leider AS, Eversole LR, Barkin ME (1985). Cystic ameloblastoma A clinicopathologic analysis. Oral surgery, oral medicine, and oral pathology.

[8] Ledesma-Montes C, Mosqueda-Taylor A, Carlos-Bregni R (2007). Ameloblastomas: a regional Latin-American multicentric study. Oral diseases.

[9] Schmid K (1956). Purification and properties of an alpha2-glycoprotein derived from normal human plasma. Biochimica et biophysica acta.

[10] Irmak S, Tilki D, Heukeshoven J (2005). Stage-dependent increase of orosomucoid and zinc-alpha2-glycoprotein in urinary bladder cancer. Proteomics.

[11] Lee SY, Lim JW, Kim YM (2001). Effect of alpha1-acid glycoprotein expressed in cancer cells on malignant characteristics. Molecules and cells.

[12] Fournier T, Medjoubi NN, Porquet D (2000). Alpha-1-acid glycoprotein. Biochimica et biophysica acta.

[13] Cheresh DA, Haynes DH, Distasio JA (1984). Interaction of an acute phase reactant, alpha 1-acid glycoprotein (orosomucoid), with the lymphoid cell surface: a model for non-specific immune suppression. Immunology.

[14] Poland DC, Garcia Vallejo JJ, Niessen HW (2005). Activated human PMN synthesize and release a strongly fucosylated glycoform of alpha1-acid glycoprotein, which is transiently deposited in human myocardial infarction. Journal of leukocyte biology.

[15] Garcia-Munoz A, Rodriguez MA, Bologna-Molina R (2012). The orosomucoid 1 protein (alpha1 acid glycoprotein) is overexpressed in odontogenic myxoma. Proteome science.

[16] Perez E, Gallegos JL, Cortes L (2010). Identification of latexin by a proteomic analysis in rat normal articular cartilage. Proteome science.

[17] Lau AT, He QY, Chiu JF (2004). A proteome analysis of the arsenite response in cultured lung cells: evidence for in vitro oxidative stress-induced apoptosis. The Biochemical journal.

[18] Bologna-Molina R, Mosqueda-Taylor A, Molina-Frechero N (2015). Differential expression of glypican-1 in ameloblastoma variants. Applied immunohistochemistry & molecular morphology : AIMM / official publication of the Society for Applied Immunohistochemistry.

[19] Dineshkumar T, Priyadharsini N, Gnanaselvi UP (2015). Evaluation and Comparison of Vascular Endothelial Growth Factor Expression between Ameloblastoma and Keratocystic Odontogenic Tumor. Journal of international oral health : JIOH.

[20] van Gool J, van Vugt H, Helle M (1990). The relation among stress, adrenalin, interleukin 6 and acute phase proteins in the rat. Clinical immunology and immunopathology.

[21] Stanciu L, Dumitrascu D, Radu D (1990). Non-specific tumoral markers in hepatocellular carcinoma. Medecine interne.

[22] Tosner J, Krejsek J, Louda B (1988). Serum prealbumin, transferrin and alpha-1-acid glycoprotein in patients with gynecological carcinomas. Neoplasma.

[23] Croce MV, Price MR, Segal-Eiras A (2001). Association of a alpha1 acidic glycoprotein and squamous cell carcinoma of the head and neck. Pathology oncology research : POR.

[24] Croce MV, Segal-Eiras A (1996). Identification of acute-phase proteins (APP) in circulating immune complexes (CIC) in esophageal cancer patients' sera. Cancer investigation.

[25] Irmak S, Oliveira-Ferrer L, Singer BB (2009). Pro-angiogenic properties of orosomucoid (ORM). Experimental cell research.

[26] Sorensson J, Matejka GL, Ohlson M (1999). Human endothelial cells produce orosomucoid, an important component of the capillary barrier. The American journal of physiology.

[27] Ligresti G, Aplin AC, Dunn BE (2012). The acute phase reactant orosomucoid-1 is a bimodal regulator of angiogenesis with time- and context-dependent inhibitory and stimulatory properties. PloS one.

[28] Bologna-Molina R, Mosqueda-Taylor A, Dominguez-Malagon H (2015). Immunolocalization of VEGF-A and orosomucoid-1 in odontogenic myxoma. Romanian journal of morphology and embryology = Revue roumaine de morphologie et embryologie.

